# Molecular genetic investigations identify new clinical phenotypes associated with BCS1L-related mitochondrial disease

**DOI:** 10.1093/hmg/ddz202

**Published:** 2019-08-22

**Authors:** Monika Oláhová, Camilla Ceccatelli Berti, Jack J Collier, Charlotte L Alston, Elisabeth Jameson, Simon A Jones, Noel Edwards, Langping He, Patrick F Chinnery, Rita Horvath, Paola Goffrini, Robert W Taylor, John A Sayer

**Affiliations:** 1 Wellcome Centre for Mitochondrial Research, Institute of Neuroscience, The Medical School, Newcastle University, Newcastle upon Tyne NE2 4HH, UK; 2 Department of Chemistry, Life Sciences and Environmental Sustainability, University of Parma, Parma, Italy; 3 Manchester Centre for Genomic Medicine, St. Mary's Hospital, Central Manchester NHS Trust, Manchester Academic Health Science Centre, Manchester, UK; 4 Institute of Genetic Medicine, Newcastle University, Newcastle upon Tyne NE1 3BZ, UK; 5 Medical Research Council Mitochondrial Biology Unit, University of Cambridge, Cambridge Biomedical Campus, Cambridge CB2 0XY, UK; 6 Department of Clinical Neurosciences, Cambridge Biomedical Campus, Cambridge CB2 0QQ, UK; 7 Renal Services, Newcastle upon Tyne Hospitals NHS Foundation Trust, Newcastle upon Tyne NE7 7DN, UK; 8 NIHR Newcastle Biomedical Research Centre, Newcastle upon Tyne NE4 5PL, UK

## Abstract

*BCS1L* encodes a homolog of the *Saccharomyces cerevisiae* bcs1 protein, which has a known role in the assembly of Complex III of the mitochondrial respiratory chain. Phenotypes reported in association with pathogenic *BCS1L* variants include growth retardation, aminoaciduria, cholestasis, iron overload, lactic acidosis and early death (GRACILE syndrome), and Björnstad syndrome, characterized by abnormal flattening and twisting of hair shafts (*pili torti*) and hearing problems. Here we describe two patients harbouring biallelic variants in *BCS1L*; the first with a heterozygous variant c.166C>T, p.(Arg56^*^) together with a novel heterozygous variant c.205C>T, p.(Arg69Cys) and a second patient with a novel homozygous c.325C>T, p.(Arg109Trp) variant. The two patients presented with different phenotypes; the first patient presented as an adult with aminoaciduria, seizures, bilateral sensorineural deafness and learning difficulties. The second patient was an infant who presented with a classical GRACILE syndrome leading to death at 4 months of age. A decrease in BCS1L protein levels was seen in both patients, and biochemical analysis of Complex III revealed normal respiratory chain enzyme activities in the muscle of both patients. A decrease in Complex III assembly was detected in the adult patient’s muscle, whilst the paediatric patient displayed a combined mitochondrial respiratory chain defect in cultured fibroblasts. Yeast complementation studies indicate that the two missense variants, c.205C>T, p.(Arg69Cys) and c.325C>T, p.(Arg109Trp), impair the respiratory capacity of the cell. Together, these data support the pathogenicity of the novel *BCS1L* variants identified in our patients.

## Introduction

Mitochondrial diseases encompass a heterogeneous group of genetic defects characterized by a dysfunction in mitochondrial energy metabolism. Clinically, defects in the mitochondrial oxidative phosphorylation (OXPHOS) system that consists of four respiratory chain enzymes (CI-CIV) and the ATP synthase (CV) manifest either within single organs or as multiorgan syndromes. The involvement of both the nuclear and mitochondrial genome in the build of the OXPHOS system underpins the varied biochemical response and clinical heterogeneity seen in patients with inherited mitochondrial diseases. OXPHOS deficiencies can be recognized as isolated events affecting individual respiratory chain complexes, or more commonly occurring as combined defects, usually affecting complexes containing mitochondrially encoded OXPHOS subunits (CI, CIII, CIV and CV).

In comparison to single enzyme deficiencies associated with Complex I and Complex IV caused by genetic defects in structural or accessory subunits, isolated Complex III defects are relatively uncommon. The enzyme cytochrome *bc_1_* or Complex III is a central enzyme of the respiratory chain, catalyzing the transfers of electrons from coenzyme Q to cytochrome *c* and subsequently forcing protons to mitochondrial intermembrane space via the Q cycle mechanism (1). Complex III functions as a homodimer (CIII_2_) that consists of 11 polypeptide subunits, one of which is encoded by the mitochondrial genome and remaining 10 are nuclear encoded. Mutations in the mitochondrially encoded structural subunit of Complex III, the cytochrome *b* gene (*MT-CYB*), have been largely associated with isolated mitochondrial myopathy and exercise intolerance. However, in recent years, the wide use of next-generation sequencing (NGS) technologies enabled the discovery of a number of variants in nuclear encoded mitochondrial Complex III structural subunits (*CYC1*, *UQCRB*, *UQCRQ*, *UQCRC2*) and assembly factors (*BCS1L*, *LYRM7*, *TTC19*, *UQCC2*, *UQCC3*) (reviewed in 2).

Pathogenic *BCS1L* gene variants are the most common cause of Complex III deficiency, and to date, over 100 different variants in *BCS1L* have been reported on ClinVar (https://www.ncbi.nlm.nih.gov/clinvar/). The *BCS1* homolog ubiquinol-cytochrome *c* reductase complex chaperone (*BCS1L*) encodes for a highly conserved yeast homolog of *bcs1* that facilitates the last step of Complex III assembly (3). BCS1L aids the insertion of the Rieske Iron-Sulphur protein, encoded by *UQCRFS1* into the pre-Complex III_2_ dimer (Supplementary Material, Fig. S1). This is an essential step in Complex III maturation and enables the enzyme to become catalytically active. The spectrum of clinical phenotypes that underlie *BCS1L* variants ranges from severe GRACILE syndrome (growth retardation, aminoaciduria, cholestasis, iron overload, lactic acidosis, early death) (4–6) to multisystemic Complex III deficiency characterized by proximal tubulopathy, liver failure and/or encephalopathy (7–9) and mild Björstand syndrome with sensorineural hearing loss and brittle hair condition known as *pili torti* (10). Understanding the pathogenicity of different *BCS1L* variants has been challenging, and poor phenotype-genotype correlations make it difficult to establish a genetic diagnosis. Although it remains to be determined why *BCS1L* variants exert such a varying spectrum of clinical phenotypes, patterns in specific amino acid substitutions and positioning within the BCS1L structure and tissue-specific manifestation of BCS1L may provide additional clues on how to improve phenotype-genotype correlations.

Here, we report two very different clinical cases of two unrelated patients with underlying pathogenic variants in the *BCS1L* gene. The individual carrying the compound heterozygous variant c.166C>T, p.(Arg56*) and c.205C>T, p.(Arg69Cys) in *BCS1L* manifested with an adult-onset aminoaciduria and phosphaturia consistent with a renal Fanconi syndrome, seizures, bilateral sensorineural deafness and learning difficulties. In contrast, the individual carrying a homozygous c.325C>T, p.(Arg109Trp) variant presented with developmental delay, persistent lactic acidosis, nephrocalcinosis, liver dysfunction and early death. BCS1L protein levels were decreased in both patients, and the activities of respiratory chain complexes were normal in muscle. A negative OXPHOS phenotype in the yeast ∆*bcs1L* null mutant was completely rescued by expressing the wild-type human *bcs1L* gene, but not with the *bcs1L*^R109W^ or *bcs1L*^R69C^ mutant genes mimicking the patients’ variants. These data provide functional evidence that supports the pathogenic effects of the *BCS1L* variants identified in our patients.

## Results

### Clinical case reports

#### Patient 1

This white British man (Supplementary Material, Fig. S2A), born to non-consanguineous parents, was referred at the age of 32 years for the investigation of biochemical disturbances including a metabolic acidosis, a raised serum alkaline phosphatase and a low serum phosphate. He has a significant past medical history of learning difficulties, seizures and bilateral sensorineural deafness. Family history revealed he had an older brother with a similar biochemical phenotype, with established chronic kidney disease and who also suffered from learning difficulties and epilepsy and died following a seizure aged 39 years. On examination, he was of slight build (height, 160 cm; weight, 44 kg; BMI, 17.4). His blood pressure was normal. Urine dipstick confirmed glycosuria (in the context of a normal blood glucose). Serum and urine biochemistry showed hypophosphatemia and renal phosphate wasting. He had established chronic kidney disease and a mild metabolic acidosis. A screen for urinary amino acids revealed a generalized aminoaciduria consistent with a proximal tubulopathy and renal Fanconi syndrome. Table S1 shows his serum biochemistry alongside his brother’s for comparison. The patient was treated with phosphate and low-dose vitamin D supplementation. The patient remained well, with good control of seizures and no unusual features. His kidney function slowly declined over the subsequent years to the present levels of serum creatinine (296 μmol/l) and eGFR (20 ml/min/1.73m^2^) (CKD-EPI) at age 49 years (Supplementary Material, Fig. S2B). On clinical examination at age 48 years, he had low set ears, mild facial dysmorphism, normal eye movements, pale optic discs but no obvious visual impairment. He had spastic dysphonia and orofacial hyperkinesias. There was no paresis, but he had tetraspasticity with increased deep tendon reflexes with upgoing plantar reflexes on the lower extremities. Romberg test result was negative, but he had a spastic-ataxic gait. He had learning difficulties. Brain MRI showed changes consistent with generalized involution (Supplementary Material, Fig. S2C), and renal ultrasound scans showed bilateral medullary nephrocalcinosis and a few small cortical cysts.

**Table 1:** Clinical, molecular and biochemical features of patients carrying *BCS1L* variants

	**Patient 1**	**Patient 2**
**Age of onset/age at last follow-up**	30 years/49 years	Birth/4 months^a^
**Sex**	Male	Female
**Family history**	Family 1—Unrelated parents, brother with learning difficulties, epilepsy and chronic kidney disease (deceased)	Family 2—Consanguineous parents, family history of unexplained childhood death
**Country of origin**	UK—Caucasian	UK—Bangladeshi
**Clinical presentations**	Learning difficulties, renal Fanconi syndrome (including aminoaciduria, phosphaturia, proximal renal tubular acidosis), epilepsy, progressive chronic kidney disease, hearing loss	Growth retardation, cholestasis, persistent lactic acidosis, early death, developmental delay, nephrocalcinosis
**MRI scan**	Generalized involution	N/A
***BCS1L* variants** ^b^ **Minor Allele Frequency (GnomAD** ^c^ **and ExAc** ^d^ **)**	Compound Heterozygous c.166C>T, p.(Arg56^*^); c.205C>T, p.(Arg69Cys) 0.0008824 and 0.0001895; 0.00006363 and 0.00005766	Homozygous c.325C>T, p.(Arg109Trp); c.325C>T, p.(Arg109Trp) 0.00004242 and 0.00006594
**PolyPhen-2** ^e^	Possibly damaging	Possibly damaging
**SIFT** ^f^	Deleterious	Deleterious
**CADD** ^g^	31	31
**CI-CIV respiratory enzyme activities**	Normal OXPHOS activities in muscle	Normal OXPHOS activities in muscle

a ^a^Age at death.

b ^b^Reference sequence NM_004328.4 and NP_004319.1.

c ^c^Genome Aggregation Database (gnomAD).

d ^d^Exome Aggregation Consortium (ExAc).

e ^e^Polymorphism Phenotyping v2 (PolyPhen-2).

f ^f^Sorting Intolerant From Tolerant (SIFT).

g ^g^Combined Annotation Dependent Depletion (CADD).

#### Patient 2

This child was born from consanguineous Bangladeshi parents (first cousin marriage). Antenatally, fetal ultrasound scans had identified both poor fetal growth (<5^th^ centile) and evidence of oligohydramnios. She was born at 36 weeks’ gestation by spontaneous vaginal delivery. APGAR scores were 9 at 1 minute and 9 at 5 minutes, and the birth weight was 1.3 kg. There was a notable family history of paternal twins (aunt and uncle) dying within the first few weeks of life of unknown cause and two paternal male cousins who had also died at 2 weeks and 14 weeks of age of unknown cause. At 2 days after birth, the child was noted to be hypoglycaemic with a raised anion-gap metabolic acidosis, with an elevated serum lactate level (5.8 mmol/l) and a raised serum ammonia level (173 μmol/l). Biochemical investigations revealed normal acylcarnitine profile (excluding disorders of fatty acid and branched-chain amino acid catabolism). Liver function was abnormal with a conjugated hyperbilirubinemia. Treatment was commenced, including sodium bicarbonate, Dalivit, vitamin E and vitamin K. Thiamine, carnitine and alphacalcidol were also given and the child stabilized, allowing discharge from the neonatal unit at 43 days old. Genetic investigations were initiated, and karyotyping showed chromosome 46XX with a del (9) (q.21.12q21.2). Skin and muscle biopsies were performed given a suspected mitochondrial disorder. Further episodes of lactic acidosis and hypoglycaemia occurred aged 74 days. Hyperinsulinism was identified and treated with oral diazoxide and chlorothiazide. An abdominal ultrasound scan revealed bilateral nephrocalcinosis and bone biochemistry showed an elevated alkaline phosphatase, with a low PTH and serum calcium. The girl deteriorated with a paralytic ileus and an acute kidney injury from which she did not recover, and she died aged 4 months of age. Unfortunately, no clinical details were available for the affected male sibling of P2 who died soon after birth.

## Molecular genetics diagnosis of *BCS1L* variants

In order to rule out monogenic forms of renal Fanconi syndrome, molecular genetic screening for variants in *CLCN5*, *OCRL1* and *CTNS* was initially performed in Patient 1 (P1). Subsequently, the patient underwent whole genome sequencing, and biallelic compound heterozygous variants in *BCS1L* c.166C>T, p.(Arg56^*^) (previously reported as pathogenic in 11) and c.205C>T, p.(Arg69Cys) were identified (RefSeq NM_004328.4). The minor allele frequency (MAF) for both variants p.(Arg56^*^) and p.(Arg69Cys) is less than 0.01, and *in silico* pathogenicity tools predicted the c.205C>T, p.(Arg69Cys) variant to be pathogenic (Table 1). Conflicting interpretations of pathogenicity have been reported for the c.205C>T, p.(Arg69Cys) variant on ClinVar that has been described as a variant of uncertain significance—likely pathogenic (12).

Given the mitochondrial disease phenotype in Patient 2 (P2) that appeared to have hallmarks of the GRACILE syndrome associated with defects in *BCS1L* and abnormal liver function previously concomitant with MPV17-related hepatocerebral mitochondrial DNA depletion syndrome, candidate gene sequencing for variants in *BCS1L* and *MPV17* was performed on genomic DNA isolated from P2. This identified homozygous missense variants c.325C>T, p.(Arg109Trp) (RefSeq NM_004328.4) in the *BCS1L* gene. The affected male sibling of P2 also carried the c.325C>T, p.(Arg109Trp) variant. The minor allele frequency (MAF) was less than 0.01, and the c.325C>T, p.(Arg109Trp) variant was predicted to by highly pathogenic (Table 1).

**Figure 1:**
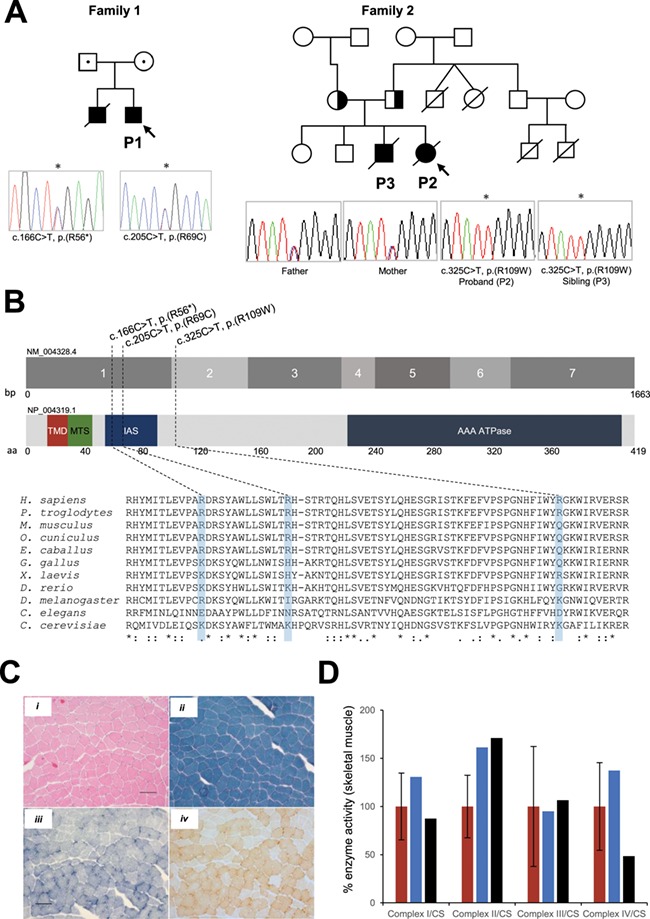
Molecular genetics, histochemical and biochemical studies of *BCS1L* patients. (**A**) Family pedigree analysis of patients carrying *BCS1L* variants and sequencing chromatographs showing the compound heterozygous variants present in P1 c.166C>T, p.(Arg56^*^) and c.205C>T, p.(Arg69Cys) and the homozygous variant c.325C>T, p.(Arg109Trp) in P2 that segregated within the family. The probands are shown by an arrow, and *BCS1L* patient variants are indicated by an asterisk. (**B**) Schematic of human BCS1L (*GeneBank: NM_04328.4, 1663bp and NP_004319.1, 419 amino acids in length*) denoting the position of the transmembrane domain (TMD), mitochondrial import sequence (MTS), import auxiliary sequence (IAS) and the AAA-ATPase domain. The positions of *BCS1L* mutations reported in the present study are indicated. Partial amino acid sequence alignments of BCS1L showing evolutionary conservation across different species. The human *BCS1L* variants are shaded in blue. (**C**) Histochemical analysis of *BCS1L* patient’s skeletal muscle (P1) subjected to (i) haematoxylin & eosin and (ii) Gomori trichrome staining showing the presence of some ragged-red fibres in the subsarcolemmal regions. Following (iii) SDH and (iv) sequential COX staining demonstrated relatively normal COX activity in P1 muscle. The scale bar shown is 100 μm. Histochemical studies were not performed on P2. (**D**) Measurement of respiratory chain enzyme activities (CI-CIV) in mitochondria isolated from skeletal muscle of control (red) and *BCS1L* patients P1 (blue) and P2 (black). OXPHOS activities were normalized to the mitochondrial matrix marker citrate synthase. Mean enzyme activities shown for muscle controls (n = 25) are set at 100%.

*BCS1L* variants were confirmed by Sanger sequencing (Fig. 1A) and mapped to the N-terminal part of the BCS1L protein (Fig. 1B). The parents of P1 (family 1) were deceased, and therefore it was not possible to carry out segregation studies. However, *BCS1L* mutation segregation analysis of P2 (family 2) showed that both parents were heterozygous carriers for c.325C>T, p.(Arg109Trp) variant (Fig. 1A).

## Muscle histopathological diagnosis and respiratory chain enzyme activities were normal in *BCS1L* patients

Histopathological assessment of biopsied muscle tissue from P1 was essentially normal. There was a slight increase in succinate dehydrogenase (SDH) and cytochrome *c* oxidase (COX) activity around the periphery of some fibres (Fig. 1C). Gomori trichrome staining suggested that subsarcolemmal accumulations were present but not overtly ragged-red (Fig. 1C). Respiratory chain enzyme activities were measured in mitochondrial-enriched muscle homogenates isolated from P1 and P2 skeletal muscle as previously described (13). Mitochondrial respiratory chain enzyme activities (Complexes I-IV) were within normal range in both patients when compared to age-matched controls (Fig. 1D).

## Pathogenic variants in *BCS1L* cause loss of BCS1L protein

**Figure 2:**
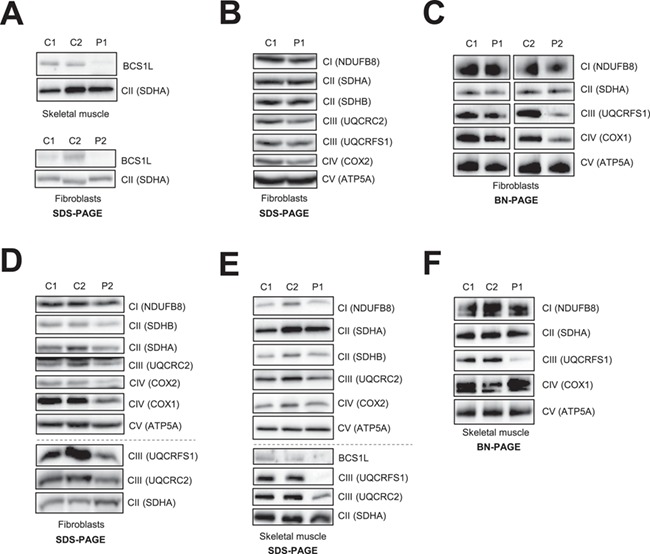
Analysis of steady-state protein levels of BCS1L and OXPHOS subunits and complexes in fibroblasts and/or muscle derived from *BCS1L* patients. (**A**) Immunoblotting of age-matched control (C1, C2) and patient (P1, P2) protein extracts against BCS1L, showing a decrease in the steady-state levels of BCS1L in muscle from P1 and fibroblasts from P2. (**B**) SDS-PAGE analysis of whole cell lysates isolated from control and P1 fibroblasts. (**C**) One-dimensional BN-PAGE analysis of DDM-solubilized mitochondrial membrane extracts of age-matched control and patient fibroblasts (P1, P2). (**D**) SDS-PAGE analysis was performed on whole cell lysates isolated from control and P2 fibroblasts. Mitochondrial extracts from control and P1 skeletal muscle homogenates were subjected to immunoblotting analysis of OXPHOS subunits and complexes following separation by (**E**) SDS-PAGE and (**F**) BN-PAGE, respectively. Muscle samples for P2 were not available for protein analysis. (B-F) Immunoblotting was performed with antibodies against individual OXPHOS subunits as indicated, and the assembly of respiratory chain complexes was assessed by using antibodies against NDUFB8 (Complex I), SDHA (Complex II), UQCRFS1 (Complex III), COX1 (Complex IV) and ATP5A (Complex V). *In all, SDHA and/or SDHB were used as loading controls*.

Western blot analysis was carried out to assess the deleterious nature of *BCS1L* variants present in P1 and P2. The steady-state levels of BCS1L protein were diminished in lysates isolated from skeletal muscle derived from P1 and fibroblasts of P2 when compared to age-matched controls (Fig. 2A). The deleterious impact of the identified genetic variants on BCS1L protein levels strongly implies that these mutations adversely affect the stability of the protein.

## The effect of *BCS1L* variants on the steady-state levels of OXPHOS subunits and complexes

To investigate the functional consequences of *BCS1L* variants on steady-state levels of OXPHOS subunits and complexes, we performed OXPHOS protein analysis on the available patient fibroblasts and muscle samples. Immunoblotting analysis confirmed that the steady-state levels of all respiratory chain complex subunits were relatively normal in fibroblasts derived from P1 when compared to controls (Fig. 2B). BCS1L has been shown to facilitate the incorporation of the UQCRFS1 subunit into mature Complex III (8) (Supplementary Material, Fig. S1). In order to assess whether there were decreased amounts of fully assembled Complex III in P1 fibroblasts, enriched mitochondrial membrane extracts were separated by one-dimensional blue native polyacrylamide gel electrophoresis (BN-PAGE) and mature Complex III was immunodetected using antibodies against UQCRFS1, whilst UQCRC2 antibodies were used to detect both mature Complex III and the pre-Complex III enzyme lacking the UQCRFS1 subunit. Consistent with the SDS PAGE analysis of P1 fibroblasts, the amounts of fully assembled Complex III and pre-Complex III were relatively normal (Fig. 2C and Supplementary Material, Fig. S3A). In addition, the amounts of OXPHOS Complexes I, II, IV and V were analyzed, showing comparable levels to age-matched control fibroblasts (Fig. 2C and Supplementary Material, Fig. S3A). Conversely, the affected P2 fibroblasts displayed a decrease in the steady-state levels of Complex III subunits UQCRC2 and UQCRFS1 and the mitochondrially encoded COX1 and COX2 subunits of Complex IV (Fig. 2D). In addition, the amounts of fully assembled Complex III enzyme immunodetected with the UQCRFS1 antibody showed a decrease in the amounts of mature Complex III in P2 fibroblasts (Fig. 2C), whilst there was a less marked assembly defect in Complex III when using antibodies against the UQCRC2 subunit (Supplementary Material, Fig. S3B). Interestingly, the amount of fully assembled Complex IV holoenzymes was also decreased in P2 fibroblasts (Fig. 2C), a finding consistent with the previously observed reduction in levels of Complex IV subunits (Fig. 2D).

The OXPHOS protein analysis was also performed on skeletal muscle homogenate that was available only from P1. Western blotting demonstrated that the steady-state levels of the Complex III subunit UQCRC2 were mildly decreased in muscle homogenates isolated from P1 (Fig. 2E). There was a marked loss of UQCRFS1 protein in both P1’s muscle (Fig. 2E) and P2’s fibroblasts (Fig. 2D), supporting the role of BCS1L in the incorporation of the Rieske Fe-S catalytic centre of Complex III. To assess the OXPHOS complex assembly in P1’s skeletal muscle, mitochondrial membrane extracts were separated by one-dimensional BN-PAGE. Consistent with the decreased levels of BCS1L and Complex III subunits (Fig. 2E), the assembly of mature Complex III was severely affected in muscle homogenates from P1 (Fig. 2F). In addition, the levels of both mature and pre-Complex III detected by UQCRC2 were markedly decreased in P1 muscle (Supplementary Material, Fig. S3C). The assembly of all the other OXPHOS complexes was normal in P1 muscle (Fig. 2F). Together, these data strongly suggest that there is a Complex III defect present in skeletal muscle from P1 and a combined OXPHOS defect in the fibroblasts of P2 affecting Complexes III and IV.

## Analysis of morphological features of the mitochondrial network in *BCS1L* patient fibroblasts

**Figure 3:**
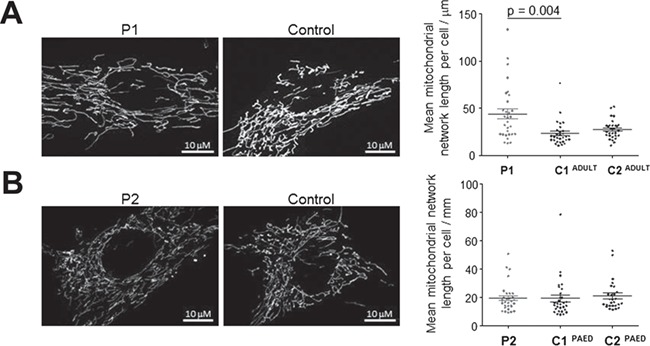
Mitochondrial network analysis of *BCS1L* patients. Patient-derived and two independent control fibroblasts were incubated with TMRM and mitochondrial network structural analysis was undertaken using MiNa on ImageJ. Mean network length was calculated, per cell, as a product of the mean branch length per cell and mean number of branches per network. (**A**) P1 fibroblasts appeared significantly hyperfused compared to C1 (*P* = 0.004), but not C2. (**B**) In contrast, P2 fibroblasts were comparable to both control cells with regards to mean network size. Results represent data from three independent experiments (n ≥ 30) and error bars represent S.E.M. One-way ANOVA statistical analysis and post hoc Dunn’s comparison were performed, and significant *P*-values have been reported.

Previous studies have reported abnormal mitochondrial morphologies in paediatric patients carrying recessive *BCS1L* variants, where patient fibroblasts showed punctate or intermediate mitochondrial tubules suggestive of a fragmented mitochondrial network (14). In order to assess the impact of *BCS1L* mutations on the mitochondrial network, confocal fluorescence microscopy studies were performed on TMRM-stained age-matched control and mutant *BCS1L* patient fibroblasts. As expected, both paediatric and adult control fibroblasts displayed a normal interconnected tubular mitochondrial network (Fig. 3). However, the total mitochondrial network length was significantly greater in P1 compared to adult control cells (Fig. 3A and Supplementary Material, Fig. S4A). In contrast, no marked differences were observed in the average mitochondrial network length between control and P2 fibroblasts harbouring the homozygous p.(Arg109Trp) variant (Fig. 3B and Supplementary Material, Fig. S4B). We hypothesize that the increased mitochondrial network length present in the cells from P1, the adult patient, may be a cell-protective compensatory mechanism that is active under metabolic stress conditions. Indeed, previous studies have suggested that mitochondrial hyperfusion is an important mechanism for the maintenance of a homogenous mitochondrial content in order to preserve efficient ATP production and resistance to apoptosis and autophagosomal degradation (15–17).

## Yeast complementation studies

**Figure 4:**
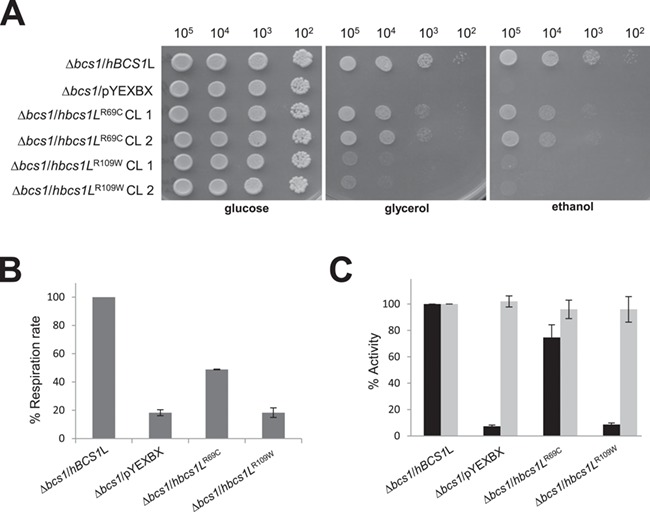
Complementation studies in yeast. (**A**) Oxidative growth phenotype. The strain ∆bcs1 was transformed with pYEX-BX plasmid carrying the wild-type human BCS1L, the mutated alleles bcs1LR69C and bcs1LR109W or the empty vector. Equal amounts of serial dilutions of cells from exponentially grown cultures (105, 104, 103, 102 cells) were spotted onto SC plates supplemented with 2% glucose, 2% glycerol or 2% ethanol. The growth was scored after 4 days of incubation at 28°C. (**B**) Oxygen consumption rates. Respiration was measured in cells grown in SC medium supplemented with 0.6% glucose at 28°C. The values observed for the bcs1L mutant cells are reported as a percentage of the respiration obtained in cells expressing the wild-type BCS1L gene. (**C**) NADH-cytochrome *c* oxidoreductase (NCCR) (in black) and cytochrome *c* oxidase (cIV)–specific activities (in grey) were recorded on a mitochondrial-enriched fraction from cells grown at 28°C in SC medium supplemented with 2% lactate (∆bcs1/BCS1L and ∆bcs1/bcs1LR69C) or 0.4% glucose and 2% lactate (∆bcs1/pYEXBX and ∆bcs1/bcs1LR109W). Values were normalized to that of BCS1L parental strain and represented as mean of at least two independent experiments.

Next we performed complementation studies in a strain of *S. cerevisiae* lacking the *BCS1* gene (yeast ortholog of human *BCS1L*), W303Δ*bcs1*, which display an OXPHOS-negative phenotype characterized by failure to grow in media containing obligatory aerobic compounds as the only carbon sources (18). The ∆*bcs1* strain was transformed with wild-type and mutated human *BCS1L* or *bcs1L*^R69C^ and *bcs1L*^R109W^ alleles cloned into the pYEX expression vector. We first evaluated the oxidative growth by spot assay analysis on SC medium supplemented with either glucose, glycerol or ethanol. As previously observed, the OXPHOS-negative phenotype could be rescued by expressing the wild-type human *BCS1L* gene (7) but not the variant *bcs1L*^R109W^, whereas the *bcs1L*^R69C^ showed an intermediate phenotype (Fig. 4A).

To better define the oxidative growth deficiency, we measured oxygen consumption, NADH-cytochrome c oxidoreductase (NCCR) and COX activity. The respiratory activity of ∆*bcs1*/*hbcs1L*^R69C^ and ∆*bcs1*/*hbcs1L*^R109W^ was, respectively, ∼50% or ∼80% less than that of parental strain (∆*bcs1*/*hBCS1*L) (Fig. 4B). Moreover, the same mutant strains displayed a specific decrease of Complex III activity (76% and 10% residual activity, respectively), whereas Complex IV (cIV) activity was normal (Fig. 4C). Together, these results support the pathogenicity of the two novel *BCS1*L variants, p.(Arg69Cys) and p.(Arg109Trp) indicating that they severely impaired the cells’ respiratory capacity.

## Discussion

We present two patients with biallelic variants in *BCS1L* with markedly different phenotypes.

The phenotype associated with P1 represents an intermediate between the very severe GRACILE syndrome and the much milder Björnstad syndrome. The overlapping features included a sensorineural deafness associated with a renal tubulopathy syndrome as well as learning difficulties and growth retardation. BCS1L-related mitochondrial disease is a useful terminology to describe patients like P1, which captures their broader phenotypes. P1 did not display features of lactic acidosis, cholestasis or iron overload. A noteworthy feature was the progressive decline in renal function such that P1 is approaching end-stage renal failure at the age of 49 years. It is likely that P1’s brother, who died before any genetic studies could be undertaken, was also affected with biallelic *BCS1L* variants, given his similar phenotype of chronic kidney disease, epilepsy and learning difficulties. The renal failure seen in P1 (and perhaps his brother) is likely to be multifactorial, but will include progressive nephrocalcinosis and likely tubulointerstitial damage secondary to mitochondrial defects (19). Indeed the renal manifestations of mitochondrial disorders are probably underrecognized and underestimated (20). P1 was of slight build. Previous reports have identified five Pakistani patients diagnosed with a Tyr301Asp variant in *BCS1L* affecting the AAA domain presenting with Björnstad syndrome. All adult family members were of short stature (152 cm, 156 cm and 161 cm) and weighing only 40 kg, 40 kg and 45 kg, respectively, with profound to severe hair loss (age 64 years, 62 years and 57 years) (21). There are similarities with P1 whose height and weight were 160 cm and 44 kg, respectively, who also exhibited hair loss.

Biochemically, we did not detect a Complex III defect in the muscle from P1; however, further analysis revealed a decrease in the levels of Complex III subunits and mature Complex III enzymes. Intriguingly, Finnish patients carrying the homozygous missense variants p.(Ser78Gly) in *BCS1L* presenting with a GRACILE syndrome had no detectable Complex III defect and no neurological findings; however, these patients had increased iron overload (6).

Here, we demonstrated that whilst BCS1L protein levels were greatly decreased in muscle from P1, there was only a mild decrease in the Complex III subunit UQCRC2 and the amount of Complex III (Figs. 2E and Supplementary Material, Fig. 3C). UQCRC2 is an early Complex III subunit and is likely to be part of a more stable pre-Complex III subcomplex, which is still detectable in P1 muscle. Previous studies have shown that BCS1L is required for the insertion of the Fe-S protein UQCRFS1 at the final stages of Complex III assembly (Supplementary Material, Fig. S1). Indeed, we observed a complete loss of UQCRFS1 protein in P1 muscle homogenate and a marked CIII assembly defect in P1 muscle (Figs. 2E and F). The absence of UQCRFS1 in P1 supports previous studies showing that BCS1L facilitates the incorporation of the Fe-S protein into the pre-Complex III.

It is possible that the two variants present in P1 p.(Arg56^*^) and p.(Arg69Cys) affect different functions of *BCS1L*. The p.(Arg56^*^) variant has been previously identified in a British patient that presented with Complex III deficiency and GRACILE syndrome (aminoaciduria, hypotonia, seizures, increased lactate) accompanied by an early death at the age of 2 days. The second variant p.(Val327Ala) identified in this patients resides in the AAA ATPase domain of BCS1L and is likely to be causative alone or in combination with the p.(Arg56^*^) (6). Perhaps, P1 is more stable due to a less deleterious nature of the p.(Arg69Cys), which is supported by our yeast studies, where complementation with a mutated human *BCS1L* allele *hbcs1L^R69C^* shows minor defect when compared to the *∆bcs1* null mutant.

In contrast to P1, P2 presented with a more classical GRACILE syndrome. However, despite a more severe clinical phenotype, Complex III enzyme activity in the muscle was normal (Fig. 1D). Conversely, we were able to show an assembly defect in OXPHOS complexes present in P2 fibroblasts, mildly affecting Complexes III + IV (Figs. 2C and D). Protein studies demonstrated BCS1L protein levels were decreased on SDS and BN-PAGE and UQCRC2 and UQCRFS1 levels were also decreased in patient fibroblasts. Interestingly, it has been suggested that the failure to incorporate the Fe-S protein into Complex III in yeast leads to instability of nascent complexes and supercomplex formation (22). Multiple OXPHOS defects have been observed in BCS1L-related mitochondrial disease (7,14). Combined OXPHOS deficiencies have been previously reported particularly in patients with severe clinical phenotypes. For instance, six Complex III-deficient patients have been identified carrying a variety of *BCS1L* mutations, all of whom died within 1 year of age (14). Furthermore, our yeast complementation data support the fact that the p.(Arg109Trp) variant produces a more severe biochemical phenotype explaining the more severe clinical phenotype (Fig. 4).

There are presently no available structures of the BCS1L N-terminal domain where the p.(Arg69Cys) and p.(Arg109Trp) missense variants are located (within the import auxiliary sequence) (Fig. 1B). Although a homology model of this domain was recently reported (23), we were unable to confidently replicate these results [a low (12.3 Å) resolution structure of the 26S proteasome regulatory subunit 6A (PDB 6EPC) was detected as a potential structural homologue using I-TASSER software (24)]. The pathogenicity of the p.(Arg69Cys) and p.(Arg109Trp) variants likely arise from the marked changes in the physicochemical properties of these residues, with the positively charged guanidinium group of arginine replaced by the smaller polar cysteine or the larger (and rigid) non-polar indole side-chain of tryptophan. Both p.(Arg69Cys) and p.(Arg109Trp) mutations are likely to disrupt the local protein environment, as well as neutralize potential stabilizing interactions.

It is noteworthy that tissue-specific expression of BCS1L may play a role in determining phenotypes (23). BCS1L is highly expressed in a wide variety of tissues, and the tissue-specific isoforms of BCS1L are likely to have an influence on the clinical phenotype by affecting different developmental pathways and tissue maintenance.

In conclusion, we have discovered and characterized new clinical phenotypes associated with *BCS1L* variants, including a patient with a *BCS1L*-related mitochondrial disease with progressive renal failure and a patient with a GRACILE syndrome harbouring a novel *BCS1L* variant. Detailed mitochondrial studies were able to reveal defects that were absent on muscle biopsy and confirm the pathogenicity of the *BCS1L* variants. Our data support the role of BCS1L in the incorporation of the Fe-S protein into Complex III, affirming *BCS1L* as a candidate disease gene in patients presenting without an obvious mitochondrial defect in muscle. Patients presenting with GRACILE syndrome or *BCS1L*-related mitochondrial disease phenotypes should be directly screened for the presence of *BCS1L* variants, or *BCS1L* variants identified by next-generation sequencing should be prioritized for further investigation.

## Materials and Methods

Written informed consent for diagnostic and research based studies was obtained from the patient 1 (P1) and the family of patient 2 (P2) in accordance with the Declaration of Helsinki protocols. Ethical approvals for P1 were obtained from the Northern and Yorkshire Research Ethics Committee (09/H0903/36) and for Genomics England from the HRA East of England—Cambridge South Research Ethics Committee.

Genomic DNA/protein was extracted from peripheral-blood lymphocytes, skeletal muscle and fibroblasts according to standard protocols.

## Molecular genetics analysis

Following informed and written consent, blood samples were obtained from P1 and P2, and DNA was extracted using standard methods. All methods were performed in accordance with the relevant ethical guidelines and regulations. For P1, sequencing of *CLCN5*, *OCRL1* and *CTNS* was performed by UKGTN. Whole exome sequencing was performed by Genomics England via the 100 000 genomes project. Confirmation of mutations was performed using Sanger sequencing, using exon-specific primers. For P2, candidate gene Sanger sequencing was performed for *BCS1L* and *MPV17.*

GnomAD (https://gnomad.broadinstitute.org/) and ExAC (http://exac.broadinstitute.org/) external variant databases were used for MAF analysis. PolyPhen-2 (http://genetics.bwh.harvard.edu/pph2/), SIFT (http://sift.jcvi.org/) and CADD (https://cadd.gs.washington.edu/) *in silico* pathogenicity tools predicting missense variants were used. Candidate variants were confirmed by Sanger sequencing.

## Cell culture

Age-matched primary control and *BCS1L* patient fibroblast cell lines were grown in high glucose Dulbecco’s modified Eagle’s medium (Sigma) containing 10% fetal calf serum, 1× non-essential amino acids, 50 U/ml penicillin, 50 μg/ml streptomycin and 50 μg/ml uridine at 37°C and 5% CO_2_ in a humidified incubator.

## Histochemical and biochemical analyses of patient muscle

Cryo-sections of skeletal muscle biopsies (10 μm) undergone standard diagnostic histochemical analyses according to previously established procedures (25). The enzymatic activities of individual mitochondrial respiratory chain complexes and citrate synthase were determined spectrophotometrically in enriched mitochondrial fractions isolated from control and patient skeletal muscle homogenates (13).

## Western blot and BN-PAGE analysis

Cellular protein extracts were prepared from control and patient fibroblasts or skeletal muscle samples and analyzed by western blotting as previously described in (26). Briefly, equal amounts of protein lysates (30–40 μg) were resolved by 12% SDS polyacrylamide electrophoresis (PAGE) and subsequently transferred on to polyvinyl difluoride (PVDF) membranes.

BN-PAGE analysis was performed to assess the steady-state levels of individual OXPHOS complexes. Mitochondrial extracts isolated from fibroblasts or skeletal muscle samples were solubilized with n-dodecyl β-d-maltoside (DDM) as described previously (26). A total of 100 μg of mitochondrial membrane protein extracts were separated by one dimensional 4% to 16% BN-PAGE and then transferred onto PVDF membranes.

Immunoblotting was carried using primary antibodies against: NDUFB8 (Abcam ab110242), SDHA (Abcam ab14715), UQCRC2 (Abcam ab14745), COXI (Abcam ab14705), ATP5A (Abcam ab14748), BCS1L (Abnova H00000617-M01), UQCRFS1 (Abcam 14 746) and Total OXPHOS Human WB Antibody Cocktail (ab110411), followed by species appropriate HRP-conjugated secondary antibodies (Dako). ECL-prime (GE Healthcare) and BioRad ChemiDoc with Image Lab software were used for detection.

## Mitochondrial network analysis

Approximately 80 000 cells were seeded into 35 mm μ-Dishes (Ibidi, 81 156) overnight. For mitochondrial network analysis, cells were incubated for 30 minutes in 5 nm TMRM (Invitrogen, T668). Facilitated by a 100× magnification oil immersion objective, an inverted multipoint scanning iSim (VisiTech) with a Ti-E microscope (Nikon) was used to capture Z-stack images of individual cells (using a 590 l filter). Triplicate experiments were undertaking using consistent microscope and laser settings (n ⩾30 cells). Images underwent Richardson-Lucy deconvolution (NIS Elements) before being processed on Fiji. Maximum intensity projections were generated and analyzed using MiNA (27). Total network length per cell was calculated by multiplying mean branch length by mean number of branches per network.

## Strains, media and generation of mutant alleles

Yeast strains used in this work were W303–1A: *MATa ade2–1 leu2–3112 ura3–1 his3–1 trp1–1 BCS1* and its isogenic *bcs1*::*HIS3* (∆*bcs1*) (18). Strains were grown in synthetic complete medium (SC) media [0.69% yeast nitrogen base without amino acids (FormediumTM, UK)] supplemented with 1 g/l drop-out mix according to Kaiser *et al*. 1994 (28), except amino acids and bases necessary to keep plasmids. Media were supplemented with various carbon sources as indicated (Carlo Erba Reagents, Milan, Italy) in liquid phase or after solidification with 20 g/l agar (FormediumTM, UK). The *bcs1*^R69C^ and *bcs1*^R109W^ mutant alleles were generated with the QuikChange II Site-Directed Mutagenesis Kit (Stratagene, La Jolla, CA, USA), using *BCS1*L cDNA cloned in the pYEX plasmid (8) as template DNA and the modified primers (base changes in bold) as follows:

BCS1R69CFw: 5′-ggttgcttagctggctcacc**t**gccacagtacccgtactcag-3′.

BCS1R69CRw: 5′-ctgagtacgggtactgtggc**a**ggtgagccagctaagcaacc-3′.

BCS1R109WFw: 5′-ccattttatctggtatt**g**ggggaaatggattcgggtagaacg-3′.

BCS1R109WRv: 5′-cgttctacccgaatccatttcccc**c**aataccagataaaatgg-3′.

After mutagenesis, sequences of inserts were verified by Sanger sequencing, and the pYEX-BX/*bcs1L*^R69C^ and pYEX-BX/*bcs1L*^R109W^ mutant constructs were used to transform the ∆*bcs1* yeast strain, using the lithium acetate method (29).

## Respiration measurement and biochemical assay in yeast

Oxygen consumption was measured at 30°C using a Clark-type oxygen electrode (Oxygraph System Hansatech Instruments England) according to (30). The activity of the respiratory complexes NADH-cytochrome *c* oxidoreductase (NCCR) and the COX (cIV) were measured spectrophotometrically on a mitochondrial enriched fraction prepared as previously described (31, 32). Statistical analysis was performed through an unpaired two-tailed t-test. Only *P*-values of less than 0.05 were considered significant.

## Funding

J.A.S. is funded by Kidney Research UK and Northern Counties Kidney Research Fund. R.W.T. is supported by the Wellcome Centre for Mitochondrial Research (203105/Z/16/Z), the Medical Research Council (MRC) International Centre for Genomic Medicine in Neuromuscular Disease, Mitochondrial Disease Patient Cohort (UK) (G0800674), the UK NIHR Biomedical Research Centre for Ageing and Age-related disease award to the Newcastle upon Tyne Foundation Hospitals NHS Trust, the MRC/EPSRC Molecular Pathology Node, The Lily Foundation and the UK NHS Highly Specialised Service for Rare Mitochondrial Disorders of Adults and Children. P.F.C. is a Wellcome Trust Principal Research Fellow (212219/Z/18/Z), and a UK NIHR Senior Investigator, who receives support from the Medical Research Council Mitochondrial Biology Unit (MC_UU_00015/9), the Evelyn Trust and the National Institute for Health Research (NIHR) Biomedical Research Centre based at Cambridge University Hospitals NHS Foundation Trust and the University of Cambridge. The views expressed are those of the author(s) and not necessarily those of the NHS, the NIHR or the Department of Health. P.G. and C.C.B. is funded by Telethon GGP15041.

## Supplementary Material

Olahova_et_al_HMG_Supplementary_data_ddz202
