# Supplementary material for: Molecular genetic investigations identify new clinical phenotypes associated with BCS1L-related mitochondrial disease
Source: Hum Mol Genet. 2019 Aug 22;28(22):3766–76. doi: 10.1093/hmg/ddz202 (PMC6935384; doi:10.1093/hmg/ddz202)
Supplement: Olahova_et_al_HMG_Supplementary_data_ddz202 [file olahova_et_al_hmg_supplementary_data_ddz202.pdf]

Supplementary Figure S1

Proposed model of the mitochondrial Complex III assembly pathway in mammals. The assembly of Complex III begins with the insertion of the mitochondrially-encoded MT CYB subunit into the inner mitochondrial membrane that is mediated by the UQCC1 and UQCC2 translational activators [1]. Heme bL is incorporated into the catalytic centre of cytochrome b followed by the binding of UQCC3 and the second heme bH [2-3]. The release of UQCC1-UQCC2 is followed by the insertion of structural subunits UQCRB, UQCRQ, UQCRC1, UQCRC2 and CYC1 and detachment of UQCC3 [4]. UQCRH and UQCR10 are incorporated and a pre-complex III2 dimer is formed [5-6]. The BCS1L assembly factor incorporates the Rieske Fe-S protein UQCRFS1 that is stabilised by the MZM1L molecular chaperone [7], followed by the insertion of the last UQCR11 subunit into CIII2 [8]. TTC19 binds the incorporated UQCRFS1 subunit of CIII2 and has a quality control function in the final steps of CIII2 maturation [9].

Supplementary Figure S2

[A] Photograph of P1 aged 49 years. [B] Progressive decline in eGFR in P1. [C] Brain MRI of P1 showing generalised involution.

Supplementary Figure S3.

Analysis of OXPHOS complexes in *BCS1L* patients. One-dimensional BN-PAGE analysis of DDM-solubilised mitochondrial membrane extracts isolated from [A] P1 and [B] P2 derived fibroblasts and age matched controls. Similarly, in [C] BN-PAGE analysis was performed on mitochondrial extracts from control and P1 skeletal muscle homogenates. fibroblasts respectively. In [A-C] the amounts of respiratory chain complexes were assessed immunologically by using antibodies against NDUFB8 (Complex I), SDHA (Complex II), UQCRC2 (Complex III), COX1 (Complex IV) and ATP5A (Complex V). In all SDHA was used as loading control.

Supplementary Figure S4

[A and B] Mitochondrial network analysis of BCS1L patients. Data underlying the calculations (mean network length = mean number of branches per network x mean branch length) presented in Figure 3. Mean number of branches per network and mean branch length were calculated, per cell, for [A] P1 and [B] P2 and two appropriate independent controls which were pooled for analysis, via MiNa on ImageJ. Results represent data from three independent experiments ($n \geq 30$) and error bars represent S.E.M. Two-tailed Mann-Whitney t-test statistical analysis was performed; $p < 0.05$ represents significant difference.

Supplementary Table S1

Table S1. Biochemical features of patients P1 and P1's sibling.

Supplementary Figure 1

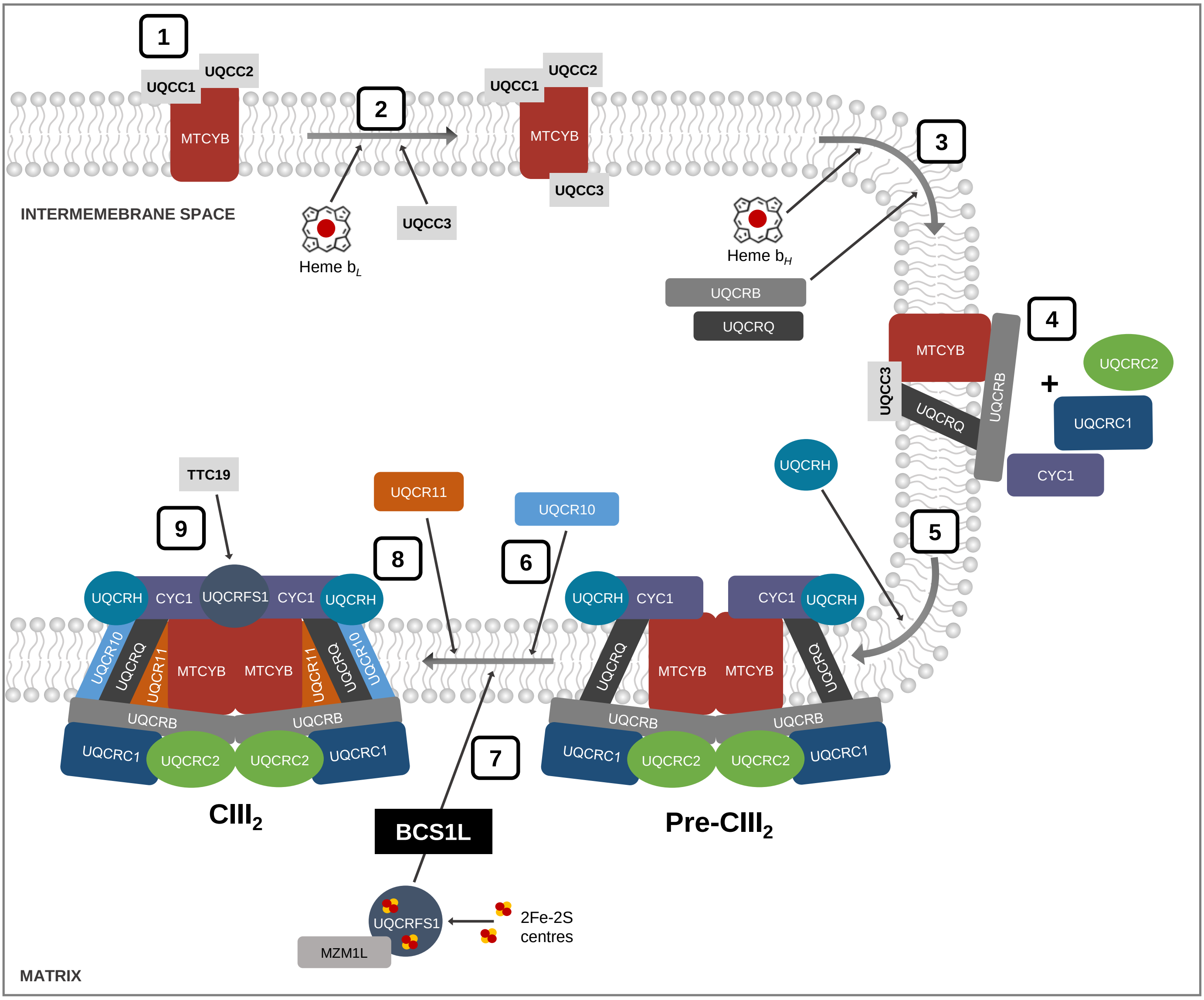

Supplementary Figure 2

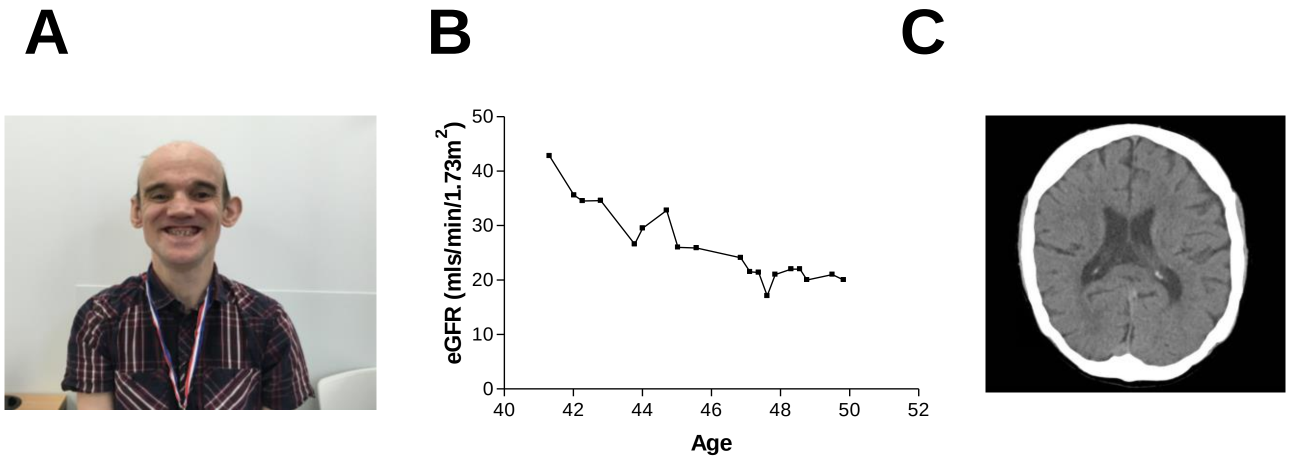

Supplementary Figure 3

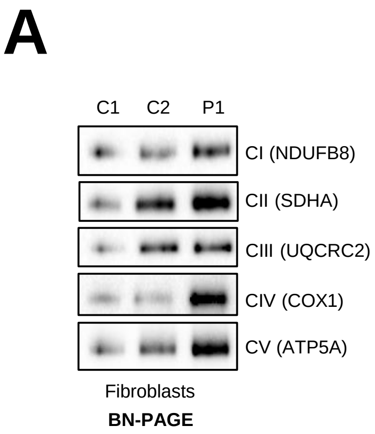

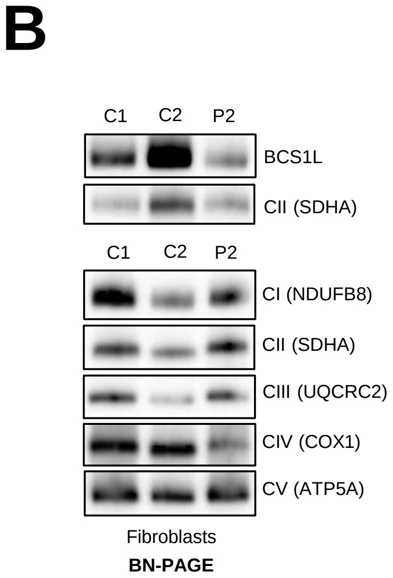

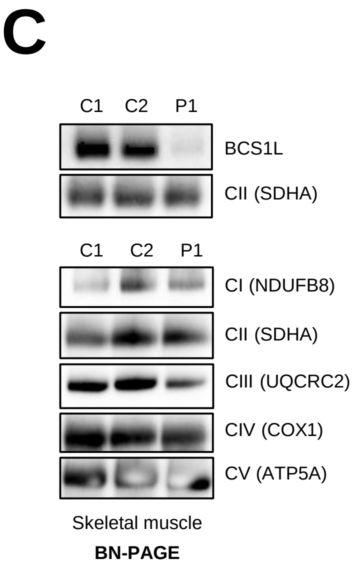

Supplementary Figure 4

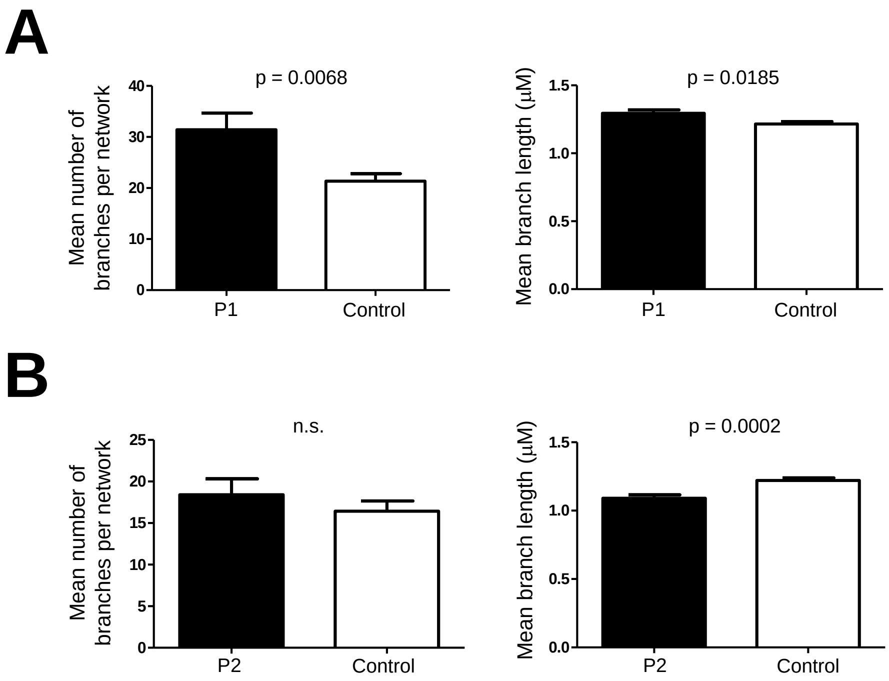

Supplementary Table 1

	P1	P1's sibling
**Plasma phosphate (mmol/L)**	0.7(↓)	1.1(↔)
**Plasma bicarbonate (mmol/L)**	17(↓)	19(↓)
**Plasma calcium (mmol/L)**	2.1 (↓)	2.06 (↓)
**Plasma creatinine (µmol/L)**	164 (↑)	210 (↑)
**Total Vitamin D (nmol/L)**	89(↔)	N/A
**Alkaline Phosphatase (IU/L)**	170(↑)	128(↑)
**Urine pH**	7.0(↑)	N/A
**PTH (ng/L)**	20	212(↑)
**Fractional Excretion of phosphate (NR 10-20%)**	79% (↑)	N/A
